# Decellularization and an In Situ Tissue Engineering Approach in the Development of an Aortic Graft: Technological Features and Mechanobiological Studies

**DOI:** 10.3390/polym17030305

**Published:** 2025-01-23

**Authors:** David Sergeevichev, Vladislav Fomenko, Elena Chepeleva, Elena Kuznetsova, Andrey Vaver, Maxim Zhulkov, Maria Vasiliyeva

**Affiliations:** 1 E.Meshalkin National Medical Research Center, Ministry of Health of the Russian Federation, 15, Rechkunovskaya Str., Novosibirsk 630055, Russia; e_chepeleva@meshalkin.ru (E.C.);; 2 N.N. Vorozhtsov Novosibirsk Institute of Organic Chemistry, Siberian Branch of Russian Academy of Sciences, 9, Lavrentiev Ave., Novosibirsk 630090, Russia; chemistnsk@yandex.ru; 3 Zelman’s Department of Medicine and Psychology, Novosibirsk State University, 1, Pirogov Str., Novosibirsk 630090, Russia

**Keywords:** xenograft, cardiac valve pathology, decellularization, mechanical strength, tissue engineering

## Abstract

This study presents a novel method to enhance the biocompatibility of decellularized porcine aortic segments while preserving their mechanical properties and histological structure. Detergent-decellularized aortic segments were treated with modified globular chitosan (Novochizol™) at varying concentrations (0.5%, 1%, 2%, and 3%) by sonication and subsequently subjected to mechanical testing. To further improve cell infiltration, blind-ended laser channels were created within the decellularized segments. The modified grafts were then seeded with porcine vascular interstitial cells in vitro for 7 days or implanted into the thoracic aorta of minipigs for 30 days. Histological analysis was performed at each stage of the study. Impregnation with Novochizol™ significantly increased the specific strength (from 0.97 ± 0.19 MPa to 4.99 ± 2.43 MPa) and Young’s modulus (from 0.73 ± 0.06 MPa to 14.66 ± 7.14 MPa) of the decellularized aortic segments. Histological examination confirmed the preservation of the connective tissue matrix’s morphological structure. Optimal modification conditions were identified as a 30 min sonication in a 1% Novochizol™ solution at 25 °C. A 35 ms continuous laser treatment was sufficient to create a 1 mm deep blind-ended channel, thereby promoting the seeding of vascular interstitial cells within the acellular graft, as confirmed by implantation in minipigs.

## 1. Introduction

Heart valve pathology requiring surgical intervention is a common cardiovascular condition affecting people of all ages. Mechanical and biological valve prostheses each have their own significant drawbacks. The former require lifelong anticoagulant therapy and are associated with an increased risk of thromboembolic or hemorrhagic events. The latter, consisting of chemically treated animal tissues, do not support the growth, regeneration, or renewal of collagen matrix or cells, and are, therefore, subject to degeneration, resulting in limited durability [1]. Donor allografts—biological valve prostheses—allow graft growth and adaptation to changing mechanical loads and are, thus, preferable, particularly in younger patients [2,3,4]. Compared to valves of artificial and xenogeneic origin, their hemodynamic characteristics are similar to the patient’s original valves, and their thrombogenicity and risk of infection are lower [5,6]. They also minimize the risk of structural degradation and the associated need for reoperation [2,6,7,8]. However, donor material is in short supply. The development of tissue-engineered valve prostheses is a promising venue to address this unmet need.

Modern achievements in tissue engineering of heart valve prostheses are developed in three main directions of biological scaffold fabrication: in vitro, in vivo, and in situ tissue engineering. Each of them has its own disadvantages, but only the last one is really suitable for practical use [9]. A cell-free, non-immunogenic scaffold with appropriate mechanical properties should become the basis for the fabrication of a valve prosthesis capable of growing and developing together with the recipient organism [10,11]. Repopulation of the cell-free scaffold by circulating cells in situ has proven to be an advantage over bioreactor technologies [12,13]. Further successful development of tissue engineering of biomaterials for cardiovascular surgery should be based on this strategy.

Extracellular matrix (ECM) scaffolds of natural origin (cell-free animal tissues) and synthetic constructs made from biodegradable polymers are the two primary types of matrices for tissue engineering [6,14]. To date, various synthetic and biological materials have been developed and studied. Different decellularized heart valves have been produced, with biological scaffolds comprising extracellular matrices made of type I collagen, Matrigel^®^, and other materials [15]. Decellularized biological matrices offer several advantages, such as mechanical anisotropy, structures similar to that of a native heart valve, and the ability to be replaced by the recipient’s own cells during recellularization [14]. In practice, ex vivo tissue engineering methods promise to restore the anatomical structure and physiological function of the replaced component of the cardiovascular system.

Optimization of decellularization methods aims to preserve tissue biomechanical properties and to reduce the residual amounts of both donor cellular material and of components of decellularizing solutions. Physical decellularization methods fail to adequately preserve the mechanical properties of cardiovascular tissues. There are different chemical decellularization methods, including enzymatic, acid–base, and detergent-based methods using surfactants. The latter are preferred because they inflict minimal damage to the extracellular matrix (ECM) fibers and effectively remove residual cellular debris and genetic material contained in cell nuclei [7,11].

Decellularization methods for the generation of cell-free ECM scaffolds must be optimized in three aspects. The first aspect concerns the decellularizing solutions that have nonspecific proteolytic and lipolytic properties, needed to destroy cells located on the surface and within the ECM. Such solutions also inflict damage to the ECM fibers, and, hence, to the scaffold’s biomechanical properties [10,16,17]. The second aspect concerns residual amounts of donor material (cell wall fragments, nuclear material) that may remain in the scaffold, leading to adverse immune reactions in the recipient. Indeed, the development of local or generalized immune-mediated cytotoxic reactions, accompanied by the degeneration of implants with fibrosis and calcification, significantly reduces the long-term, uninterrupted function of such valve prostheses [18,19]. The third aspect concerns the significant cytotoxic activity of certain high ionic strength components of decellularizing solutions with poor dissociation from the ECM. An insufficiently effective protocol for washing the ECM after decellularization would generate toxic biomaterials that are not suitable for further use [11].

One of the consequences of decellularization is a decrease in the mechanical strength of biomaterials. This is due to the specific features of the ECM of the biological tissues studied, namely the predominance of concentrically arranged fibers over longitudinal ones and the possible reduction in the number of inter-fiber connections between concentric fibers during the decellularization process [20,21,22]. While crosslinking with glutaraldehyde or epoxy compounds may restore or preserve the mechanical properties of ECMs, current methods have known drawbacks, such as an increased risk of calcification, increased stiffness, and residual toxicity of the biomaterial after treatment [1,23]. As an alternative, natural biopolymers, such as chemically modified chitosan, can be used to achieve similar results [24].

A tissue-engineered valve must be populated with functional cells present in the surrounding vascular tissue. Methods for the complete repopulation of decellularized materials have been extensively researched. In vitro expansion of endothelial cells has been successfully demonstrated in the past using a bioreactor system [25]. However, the repopulation of biomaterials with interstitial cells (fibroblasts, fibrocytes, myofibroblasts, and smooth muscle cells) remains an unsolved problem. In vivo implantation experiments involving long-term (9 months) observations have shown only minor repopulation of the matrix interstitium of the leaflet apparatus with fibroblast cells. The observed cells apparently migrated from surrounding tissues, rather than being descendants of the cellular material with which the tissue-engineered valves were seeded during manufacturing [26,27]. This finding underlies the importance of optimizing the cell-seeding method of decellularized scaffolds to increase the efficiency of cell migration into interstitial spaces. Additionally, in vitro tests must take into account the arterial pressure and mechanical loads that tissue-engineered prostheses will experience in vivo after implantation.

The main objective in seeding cell-free scaffolds is the restoration of the population of interstitial cells [21,28,29]. Since interstitial cell migration into an unmodified cell-free matrix is limited to the adventitial side (from the outside), it may be beneficial to create additional pathways for the “entry” of circulating interstitial cells and their precursors from the “inside”. To achieve this, it is necessary to modify the structure of the dense basement membrane lining the inner (facing the bloodstream) surface of vascular ECMs. Chemical treatments of the basement membrane, whether enzymatic or detergent-based, cannot be used because they cause irreversible damage to the ECM architecture that accelerates the degradation of the entire tissue-engineered graft. The preferred methods of basement membrane perforation are physical [30]. Of these, the perforation of the basement membrane using laser irradiation to form “blind-ended” channels in the ECM is an interesting option for the entry of interstitial cells into the cell-free matrix.

The goal of this study is to develop an optimized modification method to enhance the biocompatibility of decellularized porcine aortic segments while maximizing their ability to retain the original mechanical and histological characteristics of the transplanted material.

## 2. Materials and Methods

### 2.1. Preparation of Biomaterials

Porcine aortic materials were obtained from a local slaughterhouse in the cleanest possible manner. Tissues were cleaned to remove loosely attached tissues and placed in a chilled sterile physiological solution for subsequent transportation to the laboratory. Further manipulations were carried out in a sterile box in a germ-free air environment. The collected materials were cut into fragments without visible damage or natural openings.

### 2.2. Preparation of Decellularization Solutions

Sodium deoxycholate and sodium dodecyl sulfate (Applichem, Darmstadt, Germany) were weighed and dissolved in the required volume of phosphate buffer (Biolot, St. Peterburg, Russia) at room temperature with constant stirring for 1 h using an orbital shaker PSU-20 (Biosan, Riga, Latvia) until complete dissolution. The final concentration of sodium deoxycholate and sodium dodecyl sulfate was 0.5%.

### 2.3. Detergent Decellularization

Groups of samples (10 samples each) were placed in a sterile 500 mL conical flask, covered with 200 mL of decellularizing solution, and hermetically sealed with a sterile stopper. The flask was placed in an orbital thermo-shaker Unimax (Heidolf, Schwabach, Germany) and incubated for 24 h, as described previously [31,32]. The materials were then washed in the same containers used for decellularization, replacing the decellularizing solution with 300 mL of sterile phosphate buffer. After 8 washes of 12 h each, the materials were used for further studies.

### 2.4. Treatment of Decellularized Tissues with Chitosan Solutions 

Chemically modified globular chitosan (Novochizol™ (Novochizol SA, Monthey, Switzerland) was obtained as a 3% sterile stock solution in 1.5% succinic acid buffer. Solutions with lower concentrations (2%, 1%, and 0.5%) were prepared by diluting the stock solution with the required amount of sterile deionized water at room temperature. Samples (*n* = 96) were individually placed in 50 mL Falcon tubes (Axygen, Union City, CA, USA) with 50 mL of Novochizol™ solution, tightly sealed with screw caps, and incubated in an ultrasonic bath (300 W, 35 kHz) in a water bath at a temperature of 25 to 40 °C for various time intervals (from 5 to 30 min). A complete list of the solutions used, the duration of ultrasound exposure, and the temperature conditions is given below (Table 1).

### 2.5. Tensile Testing of Biomaterials 

Tensile strength tests were conducted on 10 samples after decellularization and on 5 samples per group which were subsequenty treated with Novochizol™. Rectangular sections of samples (9 × 28 mm) were cut from blanks using a standard puncher. Thickness was measured in triplicates using an electronic digital micrometer. Samples were stretched in the longitudinal direction relative to the blood flow axis, using a tensile testing apparatus (ESM-303 (Mark-10, Copiague, NY, USA)), at a rate of 20 mm/min. Specific strength and Young’s modulus were calculated for each collected sample fragment. Young’s modulus was determined in the elastic deformation region of the “stress–strain” curve.

### 2.6. Selection of Laser Processing Modes for Decellularized Matrices 

The material was processed using a laser cutting device for sheet biological materials (“Melaz-Cardio” (Institute of Laser Physics SB RAS, Novosibirsk, Russia)). When selecting irradiation modes to obtain blind-ending channels in the aortic tissue, the irradiation duration varied within a range of 30–50 ms. For each experiment, it was necessary to obtain blind-ended channels with a diameter of 100–400 μm, penetrating into the material to a depth of at least 200–500 μm. When manufacturing a prototype of a tubular aortic graft, the distance between the irradiation points was 2 mm. The irradiation duration was 35 ms. Before laser processing, the graft was turned inside out, its ends were fixed, and it was flattened to obtain a flat surface, which was then processed.

### 2.7. Scanning Electron Microscopy

After laser processing, the samples were dried and coated with a conductive carbon layer 7–10 nm thick using a carbon thermal sputtering device (GVC-3000 (KYKY Technology Co., Beijing, China)). The study was performed using a WIN SEM A6000LV microscope (KYKY Technology Co., Beijing, China) using a secondary electron detector at an accelerating voltage of 20 kV and an electron beam current of 120 μA.

### 2.8. In Vitro Cellular Seeding of Obtained Materials

Porcine aortic tissue samples treated with Novochizol™ were seeded with porcine vascular interstitial cells (VICs) under static conditions. To obtain VICs, native aortic tissue obtained before the decellularization stage was mechanically minced into 1–2 mm^3^ pieces and then incubated with a 0.2% solution of type IV collagenase (Worthington Biochemical Corporation, Lakewood, NJ, USA) for 24 h at 37 °C. The fermented tissue was repeatedly and thoroughly resuspended with a pipette, and then coarse filtration was performed using a 40 μm cell-strainer (Becton Dickinson, Franklin Lakes, NJ, USA). An equal volume of phosphate buffer (Biolot, St. Peterburg, Russia) was added to the obtained cell suspension, which was centrifuged at 300 g for 5 min. The cell pellet was then resuspended in DMEM medium supplemented with 15% FBS, 2 mM L-glutamine, and 100 U/mL penicillin/streptomycin (all Biolot, St. Peterburg, Russia) and placed in a culture dish treated with poly-D-lysine (Corning, Corning, NY, USA). Cells were cultured at 37 °C and 5% CO_2_. The culture medium was changed every 2–3 days. Cells were passaged after reaching confluence and used for experiments within 2–5 passages. For the recellularization of cell-free porcine aortic samples, 20 μL of a suspension containing 50,000 VICs in DMEM culture medium was applied with a Microfine syringe with a 30G needle (Becton Dickinson, Heidelberg, Germany) to the inner surface and into the laser channels of a 1 × 1 cm sample. Further culturing of samples with cells was carried out in DMEM supplemented with 15% FBS, 2 mM L-glutamine, and 100 U/mL penicillin/streptomycin at 37 °C and 5% CO_2_ for 7 days. To label the cell culture with green fluorescent protein (GFP), a lentiviral vector pGpur carrying the GFP gene under the control of the CMV promoter was used. Viral particles were produced in HEK293FT cell culture according to a previously described protocol [33]. One day before transduction, cells were seeded in gelatin-coated wells of 4 cm^2^ culture plates at a density of 50 × 10^3^ cells/well (approximately 60% confluence). One hour before transduction, the growth medium was changed to fresh medium (1 mL), and hexadimethrine bromide Polybrene (Sigma-Aldrich, Burlington, MA, USA) was added to a final concentration of 4 μg/mL. Then, 100 μL of medium with viral particles was added. Sixteen hours after transduction, the medium was changed to fresh medium. GFP fluorescence in cells transduced with the lentivirus pGpur was observed 48 h after transduction.

### 2.9. Implantation of Tissue-Engineered Vascular Grafts in Minipigs

As a test system for experimental studies, we used castrated male laboratory minipigs of the ICG SB RAS line, weighing 57 ± 5 kg, and aged 10–14 months. 

This study was performed in line with the principles of the Declaration of Helsinki. Approval was granted by the local ethical committee of the E. Meshalkin National Medical Research Center of the Ministry of Health of the Russian Federation (approval date 20 Dec 2021, protocol 4). Animals were kept in the vivarium in accordance with the requirements of normative and regulatory documentation. Surgical interventions (*n* = 3) were performed in a specialized hybrid veterinary operating room, observing the rules of asepsis and antisepsis. Under the intubation narcosis and mechanical ventilation, with control of ECG, invasive blood pressure, and blood oxygen saturation, prosthetics of the descending aortic arch were performed using a manufactured vascular graft. Surgical access was via a lateral thoracotomy with resection of the right 5th rib. After systemic heparinization with 5000 units of heparin (Synthes, Kurgan, Russia), the subclavian artery and the thoracic aorta above the diaphragm were cannulated for temporary shunting of blood to the lower half of the body during the main stage of implantation. The thoracic aorta was clamped, and a section of the descending arch and thoracic aorta 4–5 cm long was resected, followed by replacement with a previously washed sterile vascular graft. The graft was washed sequentially in three containers with 150 mL of physiological solution for 15 min immediately before implantation. The proximal and distal anastomoses were sutured with a 5/0 monofilament polypropylene suture. After restoration of the main blood flow, the temporary aortic shunt was removed, control aortography was performed using a mobile angiograph OEC9900 (GE Healthcare, Salt Lake City, UT, USA), and heparin was inactivated by intravenous administration of a calculated dose of protamine sulfate (Ferrein, Moscow, Russia). Then, the wound was sutured layer by layer, leaving drains, which were removed the next day. For the next 30 days, the animals were placed under observation in the vivarium, where they received antiplatelet therapy (clopidogrel, 75 mg/day) and antibiotic prophylaxis. Then, the animals were removed from the experiment using super-therapeutic doses of sodium thiopental, before euthanasia, and a control aortography was performed. For histological studies, the implant material was excised as a single block with sampling of the overlying and underlying parts of the aorta.

### 2.10. Histological Studies 

For histological studies, the materials were fixed in 10% formalin in phosphate buffer (Biovitrum, St. Peterburg, Russia) for 48 h. Further processing of the materials and preparation of paraffin blocks were carried out according to the standard method. Then, 5 μm thick histological sections were prepared on a rotary microtome (HM340E (Thermo Fisher Scientific, Waltham, MA, USA)). The sections were transferred to SuperFrost Plus slides (Menzel-Gläser, Braunschweig, Germany) and dried for 1 day at 40 °C. Sections were stained with hematoxylin and eosin using reagent kits (Biovitrum, St. Peterburg, Russia) according to the manufacturer’s recommendations, and mounted under coverslips in Biomount mounting medium (Biovitrum, St. Peterburg, Russia). 

Materials of the aorta after seeding with VICs were frozen in liquid nitrogen vapor in Tissue-Tek OCT medium (Sakura Finetek, Osaka, Japan). Sections 10 μm thick were prepared using a cryomicrotome MSM-2850 (MedTechnikaPoint, St. Peterburg, Russia) and mounted on SuperFrost Plus slides (Menzel-Gläser, Braunschweig, Germany). Then, they were fixed in 96% ethanol for 10 min and stained with hematoxylin according to the standard method and mounted under coverslips in Biomount mounting medium (Biovitrum, St. Peterburg, Russia). Microscopic studies were performed using an Axioskop 40FL microscope (Carl Zeiss AG, Jena, Germany), an ADF PRO 08 camera, and ADF ImageCapture software (ADF Optics Co. Ltd., Hangzhou, China).

### 2.11. Statistical Analysis

The research results were processed using Statistica 13 software (TIBCO Software, Palo Alto, CA, USA). The normality of the distribution was checked using the Shapiro–Wilk test. The Student’s t-test was used to compare two groups. For multiple group comparisons, a one-way ANOVA analysis of variance with Dunnett’s correction was used. Data are presented as *M* ± *σ*, where *M* is the arithmetic mean, *σ* is the standard deviation, n is the sample size, and p is the statistical significance level. The critical value of the significance level was taken as 5% (*p* ≤ 0.05).

## 3. Results

**Figure 1:**
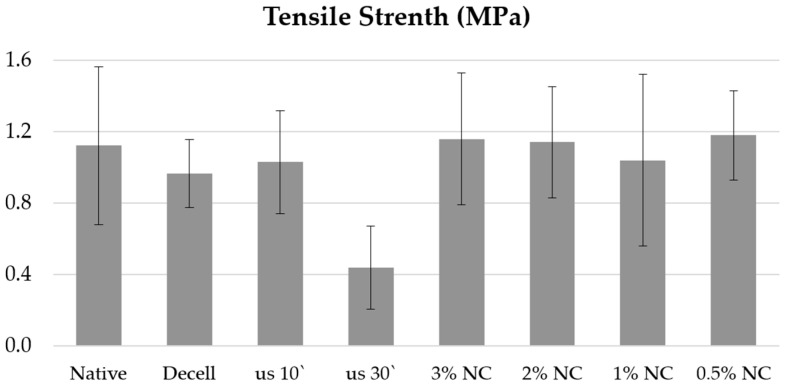
Results of preliminary tests of the specific strength of decellularized aortic samples: native—native materials, Decell—decellularized materials, us 10′—10 min ultrasound treatment in saline, us 30′—30 min ultrasound treatment in saline, and NC—incubation in Novochizol™ solution with the indicated concentration for 60 min.

At the stage of preparation for the study, a number of tests were conducted, showing that simple incubation of decellularized tissues in solutions with a Novochizol™ concentration of 0.5 to 3% on an orbital shaker for 1 h has no effect on the strength properties of the tissues. Similarly, ultrasonic treatment of samples in an isotonic solution leads only to a decrease in specific strength (Figure 1).

**Figure 2:**
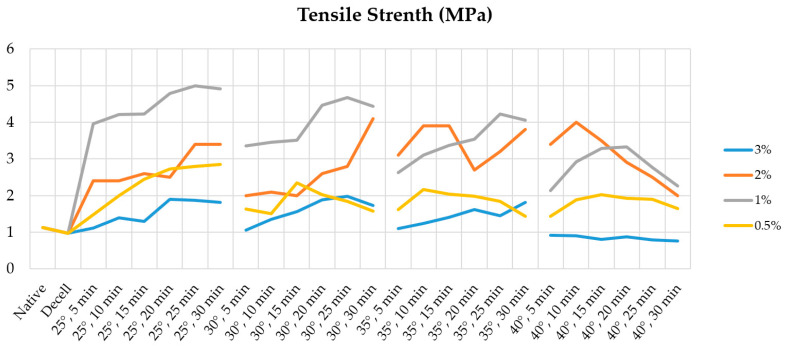
Dynamics of average strength indicators for aortic samples depending on the processing conditions: Native—native materials; Decell—decellularized materials. The legend indicates the concentrations of Novochizol™ in the solutions.

Our analysis of the results of the tensile tests showed that the specific strength of decellularized fragments of the porcine aortic wall increases from 0.97 ± 0.19 MPa to an absolute maximum of 4.99 ± 2.43 MPa when treated with 1% Novochizol™ for 25 min at 25 °C (Figure 2).

The level of increase depends on the processing conditions and the concentration of chitosan. For all studied concentrations of Novochizol™, a wave-like profile of increases in sample strength is characteristic: the strength increases with increasing processing time and reaches maximum values of 1.9 ± 0.25 MPa when treated with 3% chitosan for 20 min at 25 °C, 4.06 ± 0.19 MPa when treated with 2% Novochizol™ for 30 min at 30 °C, and 2.85 ± 1.33 MPa when treated with 0.5% Novochizol™ for 30 min at 25 °C (Figure 2). The most optimal processing time for aortic samples was a period of 25–30 min.

**Figure 3:**
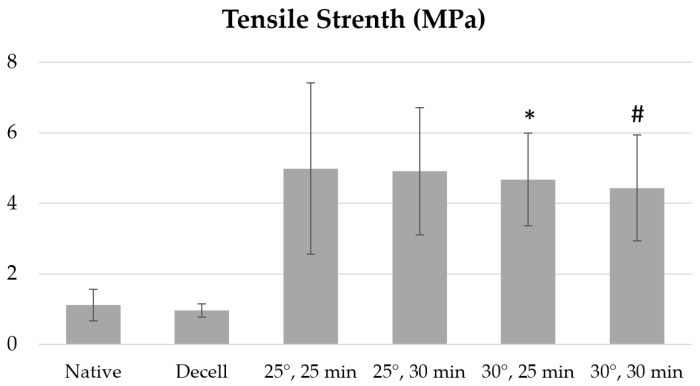
Specific strength of aortic samples treated with 1% Novochizol™ solution relative to intact materials: * *p* = 0.0206 vs. Native; # *p* = 0.0444 vs. Native; Native—native materials; Decell—decellularized materials.

The temperature conditions also had a significant impact on the specific strength of decellularized fragments of the porcine aortic wall (Figure 3).

Our data analysis shows that when the processing temperature is increased above 35 °C, the specific strength decreases regardless of the Novochizol™ concentration and processing time to minimum values of 0.76 ± 0.14 MPa when treated with 3% Novochizol™ for 30 min at 40 °C, 2.01 ± 1.25 MPa when treated with 2% Novochizol™ for 30 min at 40 °C, 2.26 ± 1.69 MPa when treated with 1% Novochizol™ for 30 min at 40 °C, and 1.65 ± 0.76 MPa when treated with 0.5% Novochizol™ for 30 min at 40 °C. Statistical analysis of the differences in specific strength of aortic samples only for a processing time of 30 min showed a significant difference both with the native material and with the decellularized one.

**Figure 4:**
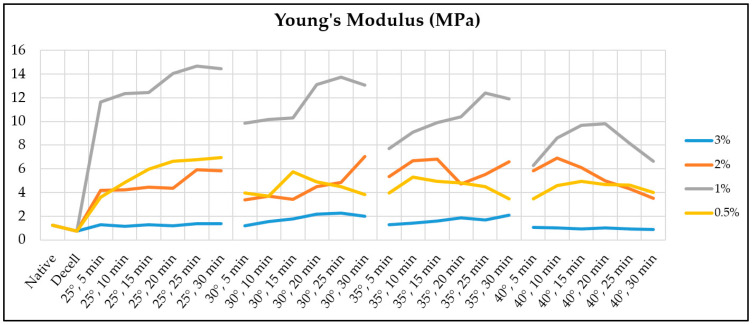
Dynamics of average Young’s modulus values for aortic samples depending on the processing conditions: Native—native materials, Decell—decellularized materials. The legend indicates the concentrations of Novochizol™ in the solutions.

The Young’s modulus of the decellularized fragments of the porcine aortic wall increases from 0.73 ± 0.06 MPa to an absolute maximum of 14.66 ± 7.14 MPa when treated with 1% Novochizol™ for 25 min at 25 °C (Figure 4).

**Figure 5:**
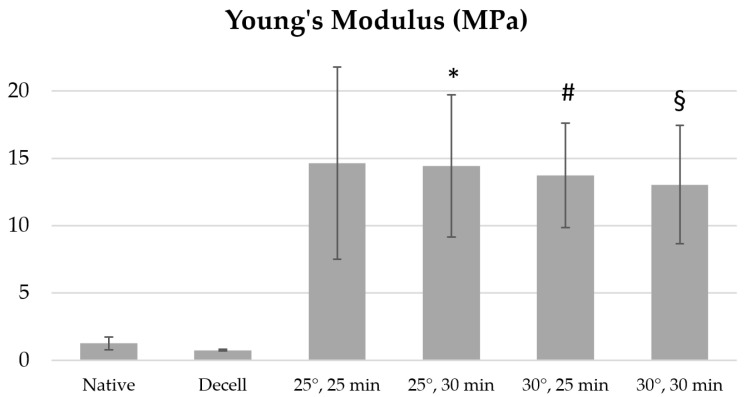
Young’s modulus values of aortic samples treated with 1% Novochizol™ solution relative to intact materials: * *p* = 0.039 vs. Native; # *p* = 0.0158 vs. Native; § *p* = 0.0306 vs. Native; Native—native materials, Decell—decellularized materials.

Young’s modulus of the remaining samples increases with increasing processing time and reaches maximum values: 2.26 ± 0.65 MPa when treated with 3% Novochizol™ for 30 min at 35 °C; 7.05 ± 3.56 MPa when treated with 2% Novochizol™ for 30 min at 30 °C; 6.93 ± 3.24 MPa when treated with 0.5% Novochizol™ for 30 min at 25 °C. Statistical analysis of the differences in Young’s modulus of aortic samples for a processing time of 30 min at 25 °C showed a significant difference both with the native material and with the decellularized one. Significant differences with the native and decellularized material were also obtained for processing times of 25 and 30 min at 30 °C (Figure 5).

**Figure 6:**
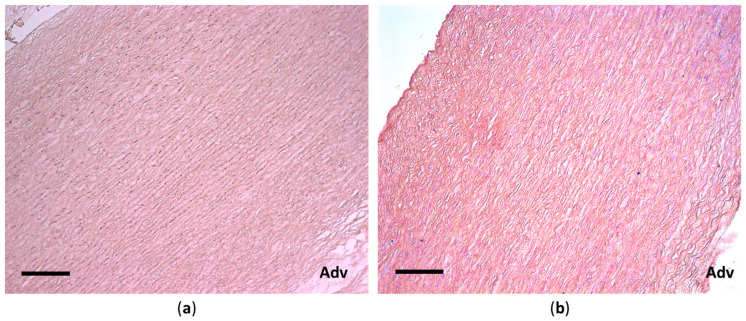
Histological structure of the porcine aortic wall before (**a**) and after (**b**) decellularization: hematoxylin–eosin staining, scale bar 200 μm. Adv—adventitial surface of the aorta.

Control histological studies of aortic materials before and after decellularization showed good preservation of the connective tissue structures and complete elimination of the cellular elements (Figure 6).

Native material is characterized by a dense, uniform packing of the main connective tissue fibers. Elastin appears as relatively thick, uniform, long, and curved strands. The aortic wall is uniformly filled with smooth muscle cells (the majority) and fibroblasts. From the intimal side, the wall is covered by endothelial cells (Figure 6a). After decellularization, voids appear in the aortic wall in places where cells were located. The waviness of elastic fibers, typical of the normal aortic wall, decreases, and the fibers moderately lose their uniform thickness. Zones of microfragmentation appear on the elastic fibers, and single zones of elastolysis are observed. Collagen fibers also partially disaggregate, and there are small areas of fiber swelling and focal homogenization (Figure 6b).

**Figure 7:**
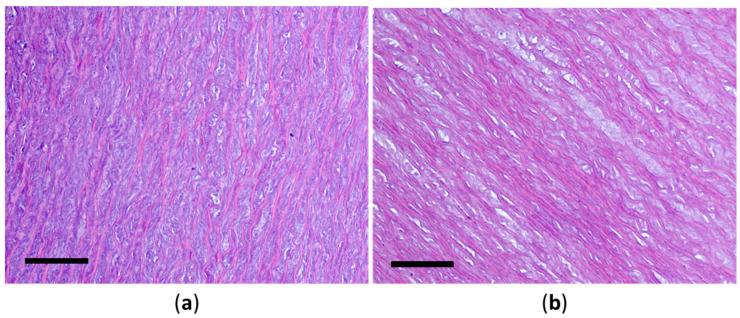
Histological structure of the porcine aortic wall after treatment with Novochizol™ (1% solution, 30 min) at a temperature of 25 °C (**a**) and 40 °C (**b**): hematoxylin–eosin staining, scale bar 100 μm.

Histological examination of the aortic fragments after chitosan treatment revealed the following findings. The temperature regime has a significant impact on the structure of the connective tissue. Increasing the temperature significantly increases collagen fiber dissociation, the number of microdamage occurrences, and the overall level of degradation. Temperature denaturation of the protein components of the collagen–elastic framework of the aorta also occurs (Figure 7).

**Figure 8:**
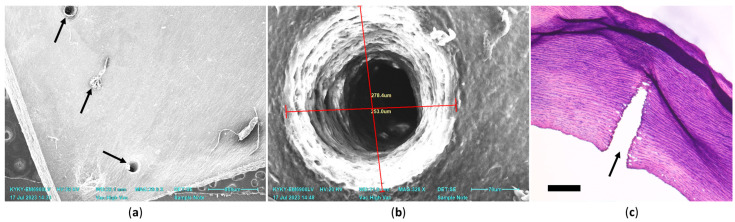
Evaluation of the size and ultrastructure of laser channels in decellularized and chitosan-treated aorta: (**a**) General view (SEM micrograph, arrows indicate channels made every 2 mm; the entrance of one channel is closed with tissue debris); (**b**) micrometry of the channel entrance on SEM micrograph (276 × 253 μm); (**c**) micrograph of the aorta after laser tunneling, transverse cryo-section; the arrow indicates the direction of laser exposure, duration 40 ms; hematoxylin–eosin staining, scale bar 500 μm.

During the preliminary selection of laser irradiation parameters for the samples, it was established that 35–40 ms of continuous operation of the laser installation is sufficient to obtain a blind-ending channel with a depth of about 1 mm, which on average is half the thickness of the aortic wall (Figure 8).

**Figure 9:**
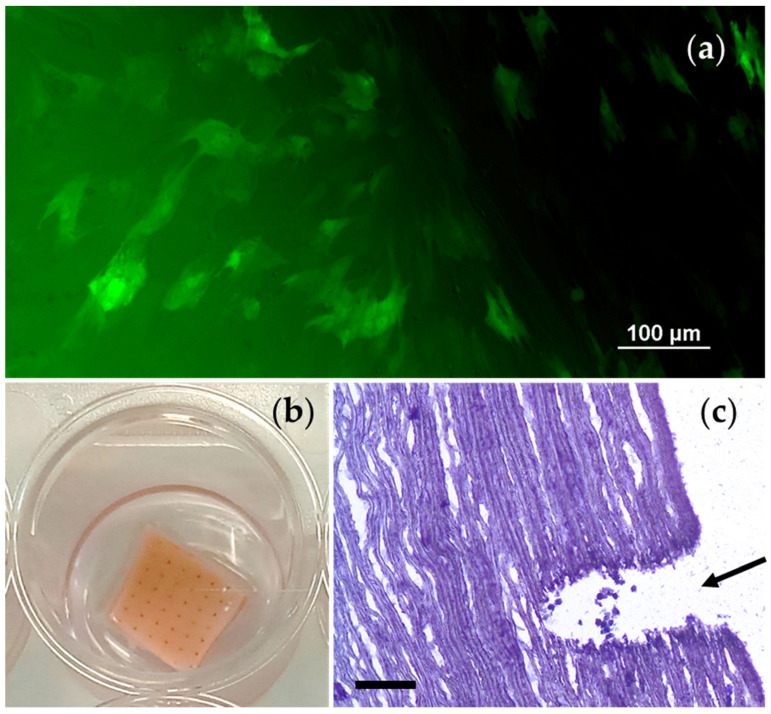
Seeding of decellularized and chitosan-treated porcine aortic samples under static in vitro conditions, observation for 7 days: (**a**) Viable GFP-labeled VICs on the surface of the treated aortic sample (scale bar 100 μm); (**b**) general view of the sample with applied VICs in a 24-well plate; (**c**) presence of VICs inside the laser channel (indicated by an arrow, 10 μm cryo-section, hematoxylin–eosin staining, scale bar 100 μm).

To assess the possibility of seeding decellularized and laser-treated biomaterials, VICs obtained from the main vessels of pigs were used. Cultivation of VICs on the surface of the samples at a rate of 50,000 cells/cm^2^ for 7 days showed the preservation of cell viability (Figure 9).

Tubular aortic prostheses for implantation into pigs were manufactured according to the following scheme:

(a) Decellularization of purified tubular fragments of porcine aorta with a diameter of 12–14 mm;
(b) Eight-fold washing in sterile phosphate buffer;
(c) Ultrasonic treatment in a 1% chitosan solution for 30 min at 25 °C;
(d) Application of blind-ended laser channels with a step of 2 mm;
(e) Decontamination in a complex alcohol solution until the moment of implantation [32].

**Figure 10:**
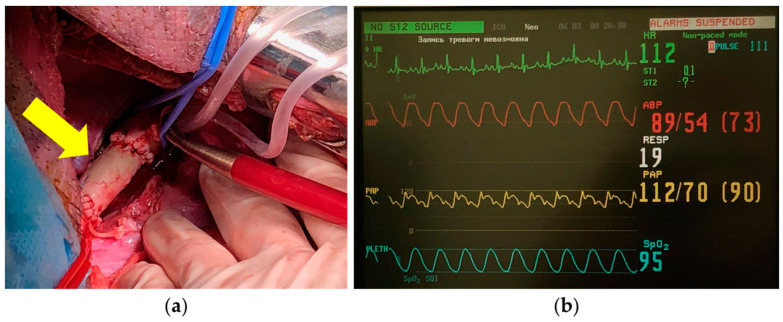
Immediate results of aortic prosthesis implantation in pigs: (**a**) the clamp is removed from the aorta and blood flow is restored; vascular prosthesis at the implantation site (indicated by a yellow arrow). (**b**) Vital signs (pulse 111–112 bpm, arterial pressure in the femoral artery 89/54 mm Hg, arterial pressure on the prosthesis 112/70 mm Hg, and peripheral blood oxygen saturation 95%).

Implantation of the developed vascular prostheses was performed in the descending aorta and thoracic aorta. To provide access to the descending aorta, a surgical technique similar to that used in human heart surgery was applied, namely a lateral thoracotomy with resection of one rib. During the operation, surgeons noted a great similarity in the techniques used between aortic prosthetics in pigs and in humans (Figure 10).

**Figure 11:**
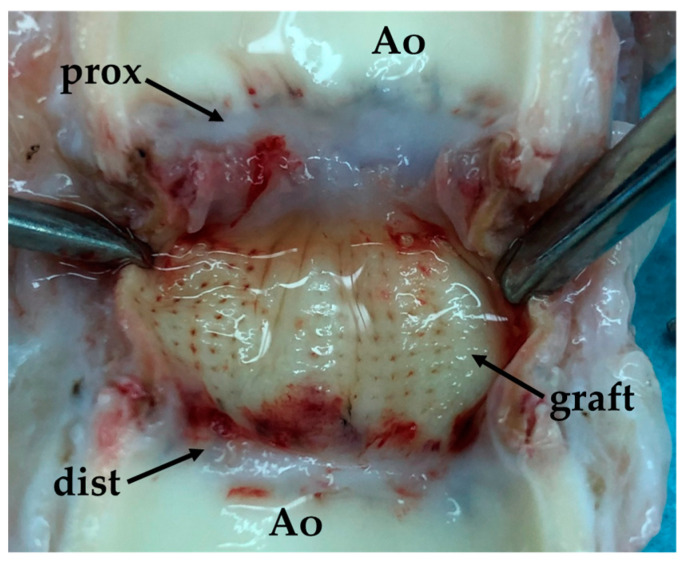
Macroscopic preparation of the developed vascular prosthesis, opened by a longitudinal incision through both anastomoses: Ao—tissues of the recipient aorta; graft—material of the implanted vascular graft; prox—proximal anastomosis; dist—distal anastomosis.

In total, three animals were operated on. In all cases, the implanted prostheses showed tightness when blood circulation was started. No cases of suture rupture were found. One animal died in the early postoperative period as a result of acute bleeding from the aortic cannulation site. The remaining animals were removed from the experiment as planned. Immediately after implantation (in the operating room) and after 30 days of observation, the animals underwent a control aortography to obtain objective data on blood flow through the implanted vascular prosthesis, to identify possible leaks and other adverse events. Analysis of the radiographs showed patency of the vascular prosthesis along its entire length. Paraprosthetic fistulas and bleeding sites were not recorded. Macroscopic examination of the removed vascular prosthesis, obtained from a decellularized porcine aorta, subjected to chitosan treatment and laser tunneling, showed the preservation of the outer and inner walls of the graft, the absence of signs of paraprosthetic leaks, thrombosis, and the beginning of epithelialization processes (Figure 11).

**Figure 12:**
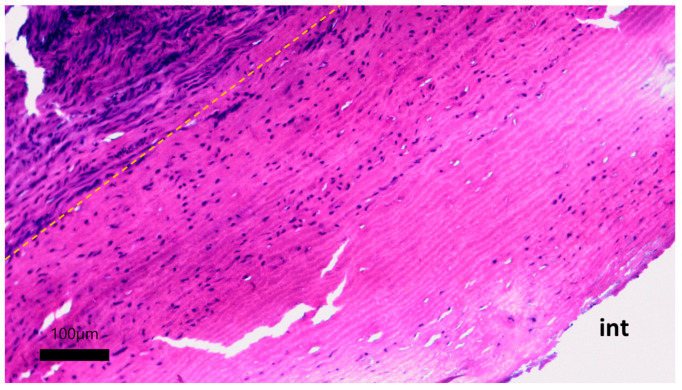
Cellular migration into the stroma of the vascular prosthesis: hematoxylin–eosin staining, scale bar 100 μm. Yellow dotted line—the boundary of the implanted vascular graft and the newly formed adventitial tissue; int—intimal side of the vascular graft.

The formation of a neointimal layer is noted directly in the anastomosis zones with further spread to the tissue of the vascular graft. Laser treatment did not have a negative effect on the permeability of the vascular graft wall for fluid. Microscopic examination of the developed vascular prostheses after 30 days of implantation in pigs revealed the initial stages of colonization of the cell-free stroma of the graft with fibroblast-like cells (Figure 12).

**Figure 13:**
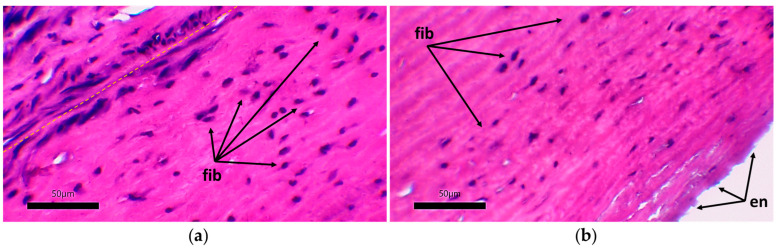
Migration of cellular elements into the acellular stroma of the vascular graft: hematoxylin–eosin staining, scale bar 50 μm. (**a**) Adventitial side of the vascular graft; (**b**) intimal side of the graft; yellow dotted line—the boundary of the implanted vascular graft and the newly formed adventitial tissue; fib—clusters of fibroblast-like cells; en—single endothelial cells.

On the micrographs, there were no zones of inflammation with foci of lymphocytic infiltration, and no signs of thrombus formation on the surface of the vascular graft were found. The tissue of the prosthesis does not differ in its structure from the original one: the packing of collagen fibers is dense, the waviness of the elastic fibers is not disturbed, and no zones of loosening were found. The stroma of the graft is populated by small fibroblast-like cells (Figure 13).

## 4. Discussion

Based on the results of prior tensometric studies, decellularized aortic fragments required improvement in mechanical properties, as decellularization negatively affected the strength of these materials. Previously, we found that chemically modified chitosan with a globular structure, Novochizol™, when interacting with decellularized tissues of the cow’s jugular vein, not only reduces the rate of their calcification but also slightly increases the strength of the materials [23]. Therefore, this processing method was optimized for use with aortic tissues. Optimization consisted of selecting the temperature conditions and incubation time of decellularized materials in solutions with different concentrations of chitosan, followed by tensometric tests, based on which the best combination of conditions was selected.

A solution with a concentration of more than 3% has too high a viscosity, which makes it unsuitable for use in the full cycle of this experiment for technical reasons: the sterilization filtration technology used through Millex-GS syringe filters does not allow for working with solutions of such a viscosity. Thus, it is possible to obtain prototypes of implants, but it is impossible to obtain them in a sterile form using this technology. Therefore, in the study, several solutions with a concentration of Novochizol™ from 0.5 to 2% were used.

An important point was the use of ultrasound: in the course of this work, the hypothesis was confirmed that it was precisely the combination of ultrasonic exposure and incubation of materials in various Novochizol™ solutions that could affect the mechanical properties of tissues.

Young’s modulus, which is used to describe the properties of materials in the region of reversible elastic deformation, from the point of view of the clinical application of the biomaterial, is a characteristic of stiffness and compliance, which is very important when creating prostheses that mimic elements of the cardiovascular system [34,35,36]. Analysis of the elastic deformation of samples allowed us to determine the most promising processing conditions, as well as to confirm the previously made conclusions.

Thus, the optimal conditions for treating decellularized aortic samples with a Novochizol™ solution to increase their strength characteristics were recognized as the use of a 1% solution, with 30 min of incubation at 25 °C. All other things being equal, lowering the temperature is a priority, since this reduces the thermal effect on the connective tissue framework of the material and the degree of its degradation, which is confirmed by histological studies. However, when comparing the microstructures of decellularized aortic fragments treated and untreated with a Novochizol™ solution, it is impossible to unequivocally determine the depth of penetration of chitosan and the nature of its interaction with the collagen–elastic framework. Existing histochemical methods for detecting aminopolysaccharides are not applicable for this analysis. Novochizol™-treated materials look subjectively somewhat denser from the outside than materials immediately after decellularization.

The problem of accelerating the migration of cells into the interstitial spaces of decellularized matrices has long been unsuccessfully solved by many scientific groups [37]. When planning this study, it was suggested that the creation of additional migration pathways for cells, such as laser channels from the basal membrane side, should accelerate the process of populating the cell-free matrix. At the same time, it was necessary to preserve the mechanical properties of the material and the tightness of the material surface [38,39,40]. The latter is necessary to exclude the penetration of blood through the wall of an implant made from the studied decellularized material.

To study the possibility of cell seeding of the obtained samples under static in vitro conditions, VICs obtained from the porcine aorta were used. VICs are a heterogeneous population of cells that include fibroblasts, fibrocytes, myofibroblasts, and smooth muscle cells [41]. These cells normally populate the stroma of connective tissue and are involved in the synthesis of the main proteins of the extracellular matrix, such as collagen and elastin [42]. The functional differences of these cells from skin fibroblasts or other localizations are the justification for their use when seeding cell-free biomaterials for the tissue engineering of vascular prostheses [43]. Despite the fact that cells remained viable even after 7 days of static cultivation, no pronounced expansion or a significant increase in the amount of cellular material were found. This confirms the conclusions of researchers about the need to create dynamic conditions for culturing fibroblasts and other VICs using specialized bioreactors to provide biophysical stimulation of cells [24,44].

To study the biocompatibility of experimental vascular grafts in vivo, the obtained samples (decellularized tubular fragments of the aorta, treated with a 1% Novochizol™ solution for 30 min at 25 °C, with blind-ended laser channels applied) were implanted into minipigs in the descending aorta and thoracic aorta for a period of 30 days. The circulatory system of pigs is closest in its hemodynamic characteristics to that of humans [45]. This feature is used in experimental studies of various implants to study the biological properties of the developed materials and implanted products. Unlike ordinary pigs, the use of minipigs has the advantage of weight and height indicators of an adult animal comparable to the average person, but with a slower growth rate and weight gain [46]. This allows the successful modelling of the conditions for implanting cardiovascular prostheses similar to the adult human body.

During macroscopic examination of the grafts removed from the animal’s body, no negative effect of the proposed experimental treatment on the preservation and permeability of the walls of the vascular prosthesis was noted. The results of histological studies of decellularized porcine aortic samples at each stage of this work showed the preservation of the morphological structure of the connective tissue matrix. The main finding was the fact of cell migration into the acellular stroma of the vascular aortic graft. It occurs not only from the adventitial side but also from the neointimal side, facing the bloodstream. In previous studies that we have conducted, we did not observe such cellular reactions. Therefore, a conclusion was made about the potentiating effect of laser tunneling of the basal membrane, which promotes the colonization of the cell-free graft with cells of the fibroblast series.

## Figures and Tables

**Table 1:** Quantitative characteristics of the biomaterial processing parameters.

Group Designation	Novochizol™ Concentration (%)	Temperature (°C)	Duration (min)
“X”, “T”, “B” ^1^	3; 2; 1; 0,5	25; 30; 35; 40	5; 10; 15; 20; 25; 30

^1^ where: “X”—chitosan concentration value, “T”—treatment temperature, and “B”—treatment duration.

## Data Availability

Additional data on this study can be provided by the authors upon special request.
